# Analysis of caudal epidurogram in single center: A preliminary study of lumbar radiculopathy management

**DOI:** 10.1097/MD.0000000000012810

**Published:** 2018-10-12

**Authors:** Bon Sung Koo, Woo Bin Kang, Jun Woo Park, So Jeong Lee, Mi Soon Lee, A Na Cho, Yang Hoon Chung, Joon Ho Lee, Yong Ik Kim, Won Seok Chae

**Affiliations:** Department of Anesthesiology and Pain Medicine, Soonchunhyang University Bucheon hospital, Soonchunhyang University College of Medicine, Gyeonggi-do, Republic of Korea.

**Keywords:** caudal anesthesia, epidural injection, fluoroscopy, low back pain, spinal stenosis, ultrasonography

## Abstract

A caudal epidural block involves placing a needle through the sacral hiatus and delivering medication into the epidural space. The procedure is safe and simple, but failure rates can be as high as 25%. The purpose of this study was to investigate the success rate of caudal epidural block by analyzing needle placement and dye flow pattern.

We retrospectively analyzed the medical records of patients who underwent caudal epidural block under spinal stenosis. A case was defined as a failure if it met at least one of the following four criteria: the epidural needle was not placed correctly inside the caudal canal; blood regurgitation or aspiration in the needle was observed; the contrast dye was injected into a blood vessel; or a large amount of the dye leaked into the sacral foramen or did not reach the L5-S1 level.

At least 1 failure criterion was observed in 14 cases (17.7%), while none of the failure criteria were satisfied in 65 successful cases (82.3%).

No matter how experienced the anesthesiologist may be, delivery of adequate therapeutic agent is not achieved in approximately 20% of cases. Therefore, we recommend fluoroscopy-guided needle placement and confirmation by radio-contrast epidurograpy as the best choice.

## 1 Introduction

Epidural injections of local anesthetic or corticosteroids via the inter-laminar, transforaminal, and caudal routes are widely accepted treatments to manage sciatica and spinal stenosis.^[1]^ Epidural injections are not only helpful for treating patients but can also help determine the future direction of treatment by providing key diagnostic information.^[2]^ Among the methods, caudal epidural block (CEB) involves placing a needle through the sacral hiatus and delivering medication into the epidural space. The procedure is safe and simple, so it is used more often than a lumbar epidural block during outpatient treatment and at primary care centers.^[2]^ However, the effects of CEB vary among patients, especially those with radiculopathy or lower back pain. These effects may vary in terms of cause and severity, raising questions about whether the methodologies used in the procedure may be problematic. A previous study^[3]^ found that the failure rate of the traditional CEB technique can be as high as 25%, even when performed by an experienced practitioner. This high failure rate may be related to inaccuracy of needle placement and failure of the therapeutic agents to flow.^[3,4]^

We wanted to compare the effects of various therapeutic methods, such as CEB and medication, in patients with spinal stenosis and radiculopathy. Therefore, we first needed to verify the accuracy of our CEB technique. Accordingly, we investigated the success rate of CEB by analyzing needle placement and the dye flow pattern in cases of CEB performed by a single experienced anesthesiologist.

## 2 Methods

This study was approved by the Institutional Review Board of our hospital (approval number: SCHBC 2018-03-002-001). We retrospectively analyzed the medical records of patients who underwent CEB due to a diagnosis of spinal stenosis in the lumbar/lumbosacral region, among outpatients who had visited our pain clinic between July 1 and December 31, 2017. All the patients’ privacy and data were maintained confidentially throughout study period. No direct contact with the patients was included in this study. Inclusion criteria included patients who had never received a previous caudal epidural injection in our hospital. Patients with a history of spine surgery were excluded from the study.

### 2.1 Procedures

The procedures were performed by a single anesthesiologist with >20 years of experience with caudal epidural injection. The patients were placed in the prone position with the spine positioning system pad on the fluoroscopic table. Betadine and alcohol were used to sterilize the surgical site, and the patient was draped so the sacral hiatus area could be exposed. The sacral hiatus was detected by palpation to identify the posterior superior iliac spine, coccyx, and sacral cornu. A skin wheal was made over the sacral hiatus using a 26-gauge needle with 2% lidocaine. A 21-gauge 10-cm Tuohy needle was inserted into the caudal canal using the blind technique without fluoroscopic guidance and advanced to <2 cm. Subsequently, a fluoroscope (OEC 9800 PLUS, GE Medical Systems, Milwaukee, WI) was used to confirm placement of the needle on a lateral view. If the epidural needle was not placed correctly inside the caudal canal, the needle was removed, and the procedure was restarted. If the needle was correctly placed, 5 mL of IOBRIX (iohexol 647 mg/mL; Taejoon Pharm, Seoul, Korea) was administered to confirm cephalad flow of the dye to the targeted level under the anteroposterior (AP) fluoroscopic view. A mixture of 3 mL of 2% lidocaine, 5 mg dexamethasone, 1500 IU hyaluronidase, and normal saline (total volume 16 mL) were injected if the dye reached the L5-S1 level. An 18-gauge Tuohy needle was reinserted under fluoroscopic guidance in cases of blood regurgitation or aspiration in the epidural needle, intravascular injection of dye, not reaching the targeted level, or a large amount leakage into the sacral foramen. The catheter was inserted through the epidural needle, and epidurography was used to confirm that the dye reached the L5-S1 level. The same therapeutic drugs were injected upon confirmation of no other complications, such as injection into a blood vessel.

### 2.2 Analysis

The fluoroscopic images were assessed by 1 pain physician who did not participate in the procedures. CEB success rate was analyzed based on medical records, including radiological images. A failed case met any one of the following four criteria: the epidural needle was not placed correctly inside the caudal canal; blood regurgitation or aspiration in the needle was observed; the contrast dye was injected into a blood vessel; or a large amount of the dye leaked into the sacral foramen or did not reach the L5-S1 level.

### 2.3 Statistics

The objective of this study was to investigate the success rate of the technique used at our medical institution. A previous study that used a similar process reported a success rate of 75%. We expected that our success rate would be significantly higher than that due to the experience of the anesthesiologist and technical advances made since the previous study. Accordingly, we assumed a success rate of 90%, and with a significance level of 0.05, statistical power of 90%, and estimated drop-out rate of 20%, the sample size required for the study was calculated to be 88, using PASS 12 version (Hintze, 2013; PASS 12. NCSS, LLC, Kaysville, UT; www.ncss.com). Data collection was completed once the target sample size (n = 88) was reached.

## 3 Results

**Figure 1:**
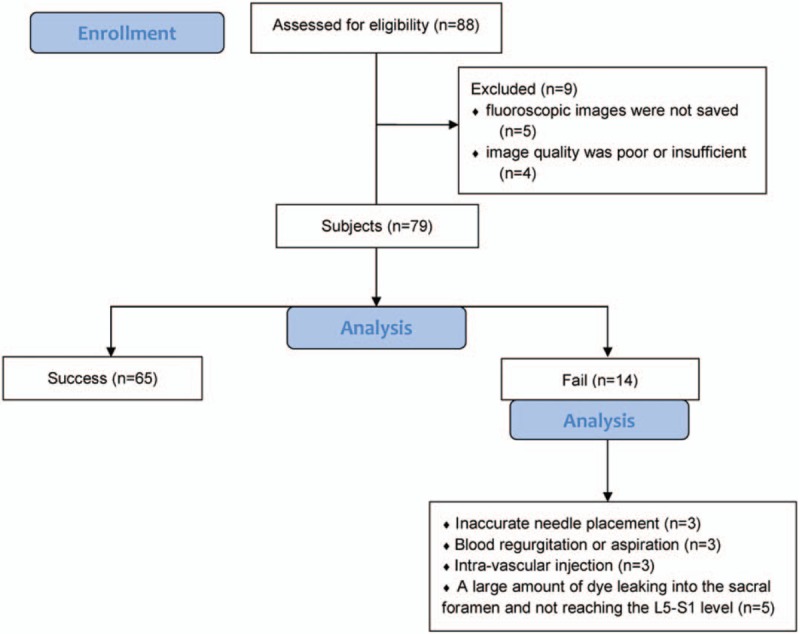
Diagram of subject enrollment and analysis.

Among the 88 patients whose data were collected, 9 were excluded from the analysis for the following reasons: fluoroscopic lateral view images were not saved after placing the epidural needle (n = 4); fluoroscopic AP view images were not saved after injecting the dye (n = 1); and image quality was poor or insufficient for analysis (n = 4). Consequently, data from 79 subjects were analyzed (Fig. 1). Patient were predominately female (70.9%) with a mean age of 67.9 ± 11.4 years (Table 1). The numbers of subjects who underwent CEB for spinal stenosis in the lumbar and lumbosacral regions were 47 (59.5%) and 32 (40.5%), respectively.

**Table 1:**
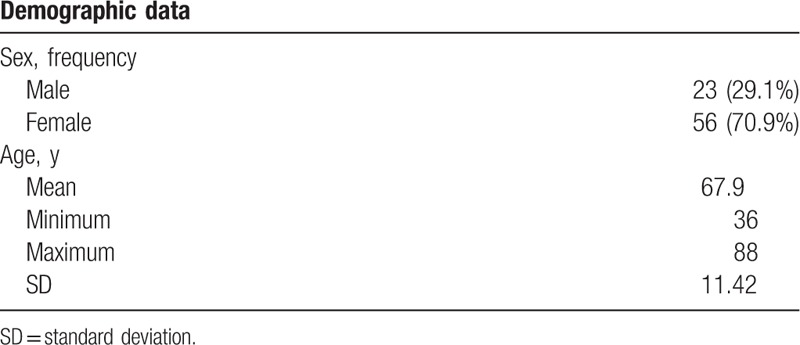
Demographic data.

**Figure 2:**
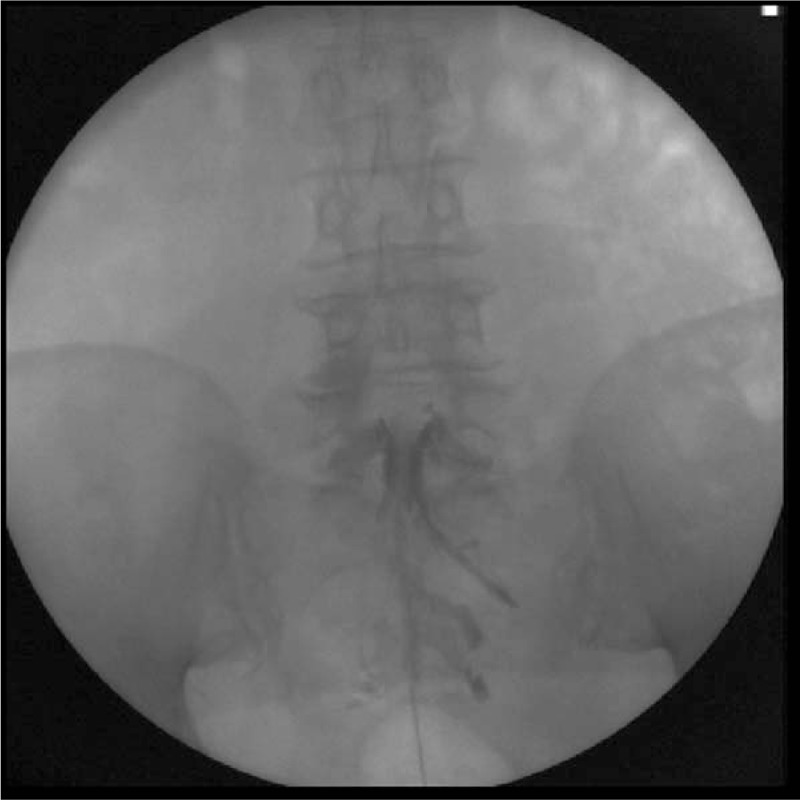
Fluoroscopic anterioposterior view showing successful migration of dye flow.

**Figure 3:**
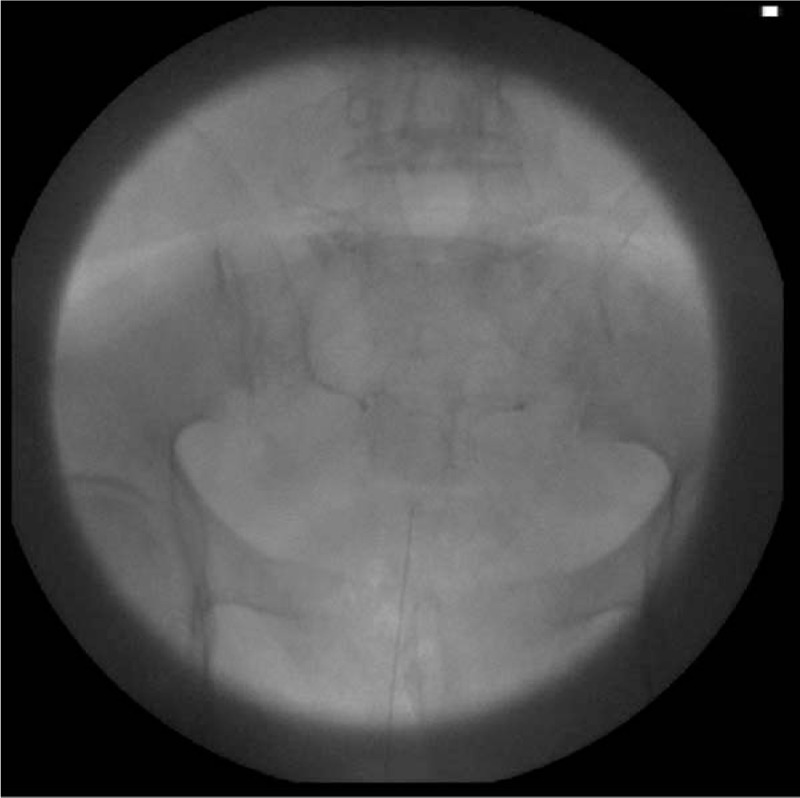
Fluoroscopic anterioposterior view showing intravascular injection of dye.

**Figure 4:**
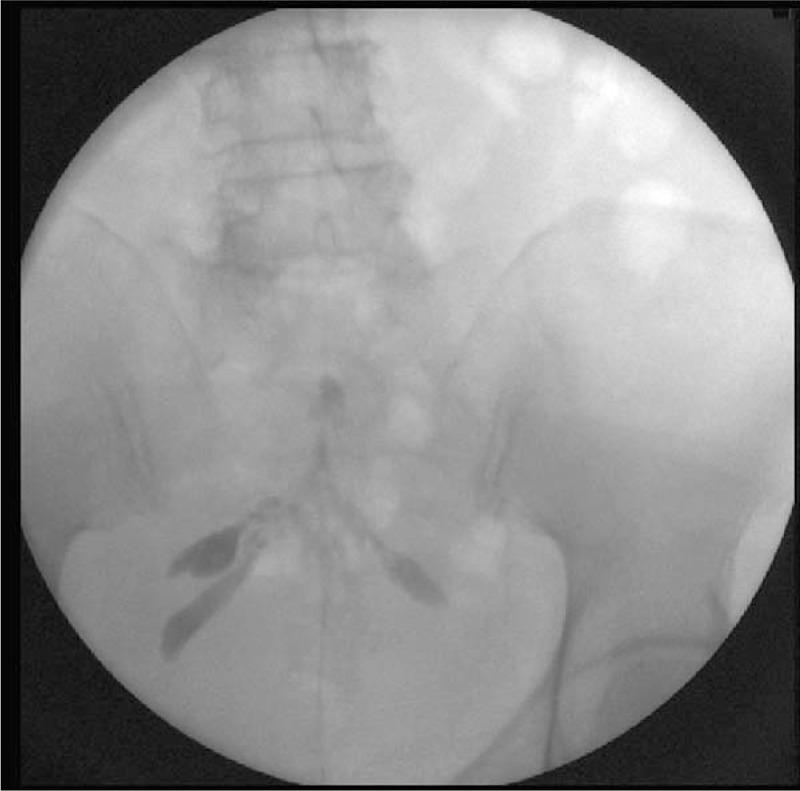
Fluoroscopic anterioposterior view showing a large amount of dye leaking into the sacral foramen.

The accuracy results for CEB revealed that at least 1 failure criterion was observed in 14 cases (17.7%), while none of the failure criteria were satisfied in 65 successful cases (82.3%) (Fig. 2). Three failed cases (3.8%) involved the epidural needle not being placed accurately inside the caudal canal as confirmed by fluoroscopic lateral view; 3 cases (3.8%) involved blood regurgitation or aspiration in the epidural needle; 3 cases (3.8%) involved dye being injected into a blood vessel, as confirmed by the fluoroscopic AP view (Fig. 3); and 5 cases (6.3%) involved a large amount of dye leaking into the sacral foramen or not reaching the L5-S1 level (Fig. 4).

## 4 Discussion

A CEB involves placing an epidural needle through the sacral hiatus to deliver medication into the epidural space. This approach is widely used for surgical anesthesia in pediatric patients, and is also popular for managing a wide variety of chronic pain in adults.^[5]^ The technique is generally known to be safe and simple, making it popular in various clinical settings. However, previous studies used to have reported on the failure rate of CEB performed using the conventional blind technique. Stitz and Sommer^[3]^ reported that when the epidural needle was placed using the blind technique without fluoroscopic guidance on 54 patients, confirmation by radiographic contrast revealed inaccurate placement of the epidural needle in 14 patients (25.9%), despite the fact that the procedure was performed by an experienced practitioner. When the failure rate was analyzed again using only the patients with a readily palpable sacral hiatus, an anatomical landmark, the results revealed a lower failure rate of 12.5%. Accordingly, the authors concluded that performing CEB under fluoroscopic guidance is the gold standard. Barham and Hiltonal^[2]^ studied 137 patients with radicular leg pain; an experienced practitioner placed the needle using a blind technique and confirmed whether the needle was placed correctly in the epidural space through lateral fluoroscopic images. The results revealed that the needle was placed correctly in 102 patients (74%). Notably, the failure rate was 26%, although it was possible to place the needle repeatedly until the practitioner was confident and satisfied that the epidural needle was correctly placed in the epidural space.

The “whoosh test,”^[6]^ which is a type of blind technique, involves using a stethoscope to listen to the midline thoracolumbar region while 2 mL of air is injected through the epidural needle. If the epidural needle is placed correctly in the caudal epidural space, a “whoosh” sound can be heard. Eastwood et al^[7]^ reported that the whoosh test is helpful for accurately placing a needle, with sensitivity of 80% and specificity of 60%. However, it may increase the risk of injecting air intravenously or an air embolism.^[8]^

A successful CEB can be confirmed not only by ensuring accurate placement of the needle in the epidural space, but also by the cephalad flow of the dye to the targeted level without complications, such as an intravascular injection. Accurate placement of the epidural needle into the caudal canal is a prerequisite for successful CEB. CEB was first introduced as a landmark-based, blind technique.^[5]^ When placing the epidural needle using a blind technique, entry into the epidural space can be achieved quickly and easily in patients with no significant anatomical variations.^[9]^ However, entry into the epidural canal may be difficult in cases where anatomical landmarks cannot be clearly identified, such as when abnormal sacral curvature or morbid obesity are present, or when deformity is present in the sacral coccygeal area due to previous trauma or birth defect.^[9]^ Doo et al^[10]^ summarized the main causes of a failed traditional technique as follows: failure to identify the sacral hiatus due to uncertain surface anatomy or anatomical variations; difficult to insert the needle through a sacral hiatus that is too narrow; and impossible to advance the needle into the sacral canal due to a small AP diameter. Kim et al^[11]^ reported that when performing a CEB using a blind technique, needle insertion into the caudal epidural space is difficult if the AP diameter at the apex of the sacral hiatus is <3.7 mm. Chen et al^[12]^ reported that ultrasound-guided needle insertion may be difficult if the AP diameter is <1.6 mm. As this was a retrospective study, information on anatomical features was not available. However, we empirically observed that in some cases the epidural needle touched or penetrated the posterior surface of the sacrum base after passing through the sacrococcygeal ligament. In such cases, there is a high probability of blood regurgitation into the needle or the dye being injected into a blood vessel. Such intraosseous placement of the needle may occur in elderly patients with osteoporosis or during nonfluoroscopic-guided injections.^[9]^

In addition to anatomical considerations, the experience and skill level of clinicians can also affect accuracy of the needle placement. Renfrew et al^[13]^ reported that there is a definite learning curve in accurately placing a needle during a caudal epidural injection. They compared the accuracy of needle placement based on experience, and found that clinicians with <10 cases of experience had a misplacement rate of 52.3%, while those with 10 to 50 and >50 cases of experience had misplacement rates of 46.6% and 38.3%, respectively. The anesthesiologist in our study had close to 30 years of experience, and as a result, the failure rate associated with needle misplacement was very low (3.8%).

If the epidural needle is accurately placed in the caudal canal, dye is injected to ensure that complications, such as intravascular injection, do not occur and that the dye flows to the targeted level. Causes of inaccurate epidural injection include abnormal sacral curvature, morbid obesity, blind injections, severe spinal canal stenosis, inadequate needle placement, and intraosseous placement of the needle.^[9]^ In our study, after confirmation of needle placement in the caudal space, a case was defined as failed if: there was blood regurgitation or aspiration in the needle; dye was injected into a blood vessel; or a large amount of dye leaked into the sacral foramen or did not reach the L5-S1 level.

An inadvertent intravascular injection should be avoided. Ergin et al^[8]^ reported on 10 patients with chronic lower back pain and radiculopathy. An epidural needle was introduced through the sacral hiatus into the caudal canal, and 2 mL of iohexol was administered after negative aspiration for blood or cerebrospinal fluid. When the final position of the needle was identified on fluoroscopic lateral view images, the needle was placed abnormally in 1 out of 10 patients. In the remaining 9 patients with accurate placement in the caudal space, real-time images were recorded in the fluoroscopic AP view during injection of 10 mL of contrast material. Of the 9 patients, intravenous leakage was detected in 4 patients. Therefore, fluoroscopic control may increase the accuracy of needle placement, but fluoroscopic guidance alone, without real-time imaging, may not be sufficient to confirm the accuracy of caudal epidural placement.

Renfrew et al^[13]^ reported venous injection in 29 of 316 procedures (9.2%), even when the epidural needle was positioned within the sacral canal and no blood was evident on the Valsalva maneuver or aspiration. Therefore, the presence of blood on the needle stylus is not a reliable indicator of venous placement of a needle. Consequently, negative blood aspiration does not guarantee that the needle is not in an epidural vein, which may collapse upon aspiration.^[2]^

The contrast agent injected into the epidural space flows to the region of least resistance, and the flow is also affected by intrinsic and extrinsic factors of the patient.^[14]^ As the patient age increases, anatomical deformation or compression may occur in patients with degenerative disease, joint disease, or spinal stenosis.^[15]^ Therefore, the dye flow pattern may be different, and the efficiency of the administered drug may decrease as the injected drug fails to reach the targeted level.^[16]^ Moreover, an atypical flow pattern may appear depending on the severity of the epidural pathology, such as epidural adhesions or fibrosis.^[10]^ Therefore, epidurography can be used to determine the degree of epidural adhesions. Jo and Jang^[16]^ reported that dye flow patterns are significantly associated with pain severity in patients with lower back pain, but that the direction of dye flow is not significantly correlated with pain laterality. We assumed that an obstruction or restriction of dye flow may be more closely associated with the patient's spinal pathology and pain severity than with the procedure itself. Finally, confirming the flow of dye or therapeutic agent by epidurography is very important.

**Figure 5:**
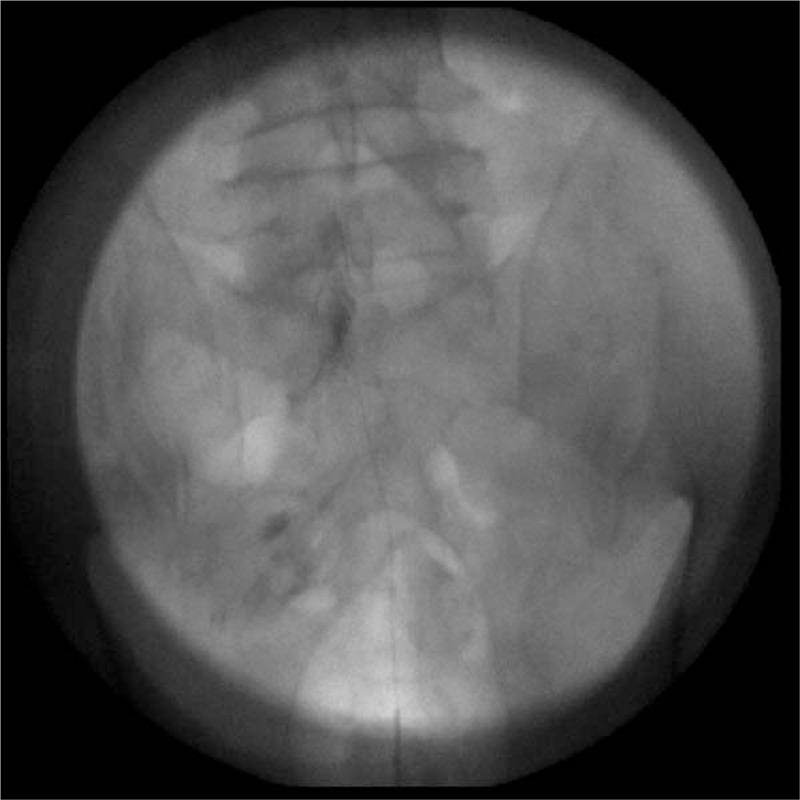
Fluoroscopic anterioposterior view showing dye flow pattern through the catheter.

We limited the study population to patients who underwent CEB for the first time because it is important to identify the extent of epidural adhesions in the initial state by epidurography, which provides diagnostic information and helps establish future treatment plans. Barham and Hilton^[2]^ assessed the cephalad spread by epidurography with a dye injection, and found that the dye reached the documented level of spinal pathology in 94% of cases. In cases with a significant obstruction of dye flow, they abandoned the caudal route and performed a lumbar inter-laminar epidural block. In the present study, a large amount of dye leaking into the sacral foramen or not reaching the appropriate level (L5-S1) was observed in 5 cases (6.3%), despite the needle being placed accurately. In these cases, we reinserted an 18-gauge epidural needle, and the catheter was inserted through the epidural needle. The drugs were injected through the catheter into the caudal canal (Fig. 5).

In this study, 5 mL of dye was used for epidurography. In previous studies,^[2–4,8,10,16]^ the volume of dye used varied from 1 to 5 mL. The decision to use 5 mL of dye, which is slightly more than that is typically used, was based on the lengthy experience of the practitioner. The rationale for this is that if the dye flow pattern does not reach the targeted level despite using a higher volume than the 2 to 3 mL typically used, then other causes, such as spinal canal stenosis or epidural space adhesions, may be suspected, and the direction of treatment can be changed accordingly. Moreover, the total volume of therapeutic agent injected into the epidural space was set to 16 mL based on the lengthy experience of the practitioner. The purpose of this was based on expectation of the adhesiolysis effect from a higher volume.

Ogoke et al^[9]^ reported that a wide range of caudal injection volumes (10–64 mL) can be used, while also stating that the volume needed to reach the L5 segment is 10 mL and to the L4 segment is 15 mL. However, injecting a high volume into the epidural space may cause retinal hemorrhage, due to increased intraocular pressure.^[17,18]^ Ogoke et al^[9]^ reported that increasing the volume does not necessarily increase spread into the epidural space or into the nerve roots, and that increasing the volume of injectate to >10 mL does not seem to improve the filling pattern.

Another method for improving the accuracy of needle placement during CEB is the use of ultrasound. Ultrasound-guided caudal block was first described by Klocke et al in 2003.^[19]^ Chen et al^[20]^ reported a 100% accuracy rate for caudal epidural needle placement into the caudal epidural space under ultrasound guidance as confirmed by contrast dye fluoroscopy. The advantages of ultrasound are that it is easy to use, it is radiation free, and can be used in virtually any clinical setting.^[21]^ Most significantly, ultrasound provides real-time and continuous needle-guiding images without radiation exposure.^[20]^ Perhaps the only disadvantage of ultrasound is the fact that it cannot provide information on to the depth of the inserted needle. Therefore, there is a risk of a dural puncture with needle advancement, so cerebrospinal fluid leakage due to a dural tear is possible.^[4,20]^ Both ultrasound guidance and the whoosh test offer the advantage of no radiation for the patient and practitioner. However, they also have the disadvantage of not being able to confirm whether the therapeutic agent was effectively delivered to the spinal pathology level.^[2]^ Consequently, fluoroscopic-guided epidurography may be necessary to confirm the dye flow pattern and injection of the therapeutic agent.

This study has several limitations. First, this study is a retrospective study of chart review. Therefore, the adequacy of the data and the omission of information are likely to have some effect. Second, fluoroscopic images assessment is done by 1 physician. However, in analyzing radiologic images, particularly in the case of a dye flow pattern analysis, there is a possibility that subjective judgments of anesthesiologist may be involved. Third, we have not evaluated the relationship between the epidurographic findings and patient outcomes. Further studied may be required to improve the above limitations.

The CEB is widely used to treat pain in cases involving spinal stenosis or radiculopathy. We analyzed the accuracy of the technique performed by a single anesthesiologist in our institution and found an 82.3% success rate. Previous studies mainly analyzed accurate needle placement during CEB. We analyzed the dye flow pattern, as well as accurate needle placement, as criteria to determine the success of CEB. In conclusion, our results indicate that adequate therapeutic agent may not be delivered in approximately 20% of cases, regardless of the anesthesiologist's experience. Therefore, we recommend, especially for those undergoing CEB for the first time, fluoroscopy-guided needle placement and confirmation by radio-contrast epidurograpy as the best choice.

## Acknowledgment

The authors thank the Senior statistician Ji Eun Moon, PhD (Department of Biostatistics, Soonchunhyang University Bucheon Hospital) for statistical consultation.

## Author contributions

**Data curation:** Jun Woo Park, So Jeong Lee.

**Formal analysis:** Joon Ho Lee.

**Resources:** Bon Sung Koo, Joon Ho Lee, Yong Ik Kim, Won Seok Chae.

**Software:** Mi Soon Lee, A Na Cho.

**Supervision:** Bon Sung Koo, Yong Ik Kim.

**Visualization:** Yang Hoon Chung.

**Writing – original draft:** Woo Bin Kang.

**Writing – review & editing:** Bon Sung Koo.

Bon Sung Koo orcid: 0000-0003-1578-6950.
